# Rational Design of Diketopyrrolopyrrole-Based Small Molecules as Donating Materials for Organic Solar Cells

**DOI:** 10.3390/ijms160920326

**Published:** 2015-08-27

**Authors:** Ruifa Jin, Kai Wang

**Affiliations:** Inner Mongolia Key Laboratory of Photoelectric Functional Materials and College of Chemistry and Chemical Engineering, Chifeng University, Chifeng 024000, China; E-Mail: Wangkai820707@126.com

**Keywords:** diketopyrrolopyrrole, electronic and optical properties, charge transport property, organic solar cells (OSCs)

## Abstract

A series of diketopyrrolopyrrole-based small molecules have been designed to explore their optical, electronic, and charge transport properties as organic solar cell (OSCs) materials. The calculation results showed that the designed molecules can lower the band gap and extend the absorption spectrum towards longer wavelengths. The designed molecules own the large longest wavelength of absorption spectra, the oscillator strength, and absorption region values. The optical, electronic, and charge transport properties of the designed molecules are affected by the introduction of different π-bridges and end groups. We have also predicted the mobility of the designed molecule with the lowest total energies. Our results reveal that the designed molecules are expected to be promising candidates for OSC materials. Additionally, the designed molecules are expected to be promising candidates for electron and/or hole transport materials. On the basis of our results, we suggest that molecules under investigation are suitable donors for [6,6]-phenyl-C_61_-butyric acid methyl ester (PCBM) and its derivatives as acceptors of OSCs.

## 1. Introduction

Solar cells have been widely considered as a next-generation clean and renewable energy resources to relieve the global energy crisis. Accordingly, various solar cells have been developed. Among them, organic solar cells (OSCs) have attracted considerable research interest because of their outstanding advantages such as low cost, light weight, easy fabrication, and flexible features [1,2,3,4,5]. Since the pioneering work of Tang on donor-acceptor thin film OSCs [6], intensive interdisciplinary efforts have been focused on improving the power conversion efficiencies (PCEs) of OSCs by using novel materials and the different device structures [7,8]. It has been demonstrated that the PCEs of the device for solution-processed polymer bulk heterojunction (BHJ) solar cells has exceeded 10% [9,10]. Unfortunately, polymeric materials suffer from batch-to-batch variation, polydispersity, indefinite molecular weight, and impurity [11]. On the other hand, solution-processed small-molecule OSCs have also attracted increasing attentions because they can deliver respectable PCEs, which are comparable to those of polymer solar cells [12,13]. These trigger the momentum for the rational design of small molecules with novel structural features used for solar cells. Small molecule OSC materials are more suitable than those of polymers because of their advantages in terms of well-defined molecular structure, accurate molecular weight, high purity, no end group contaminants, easy mass-scale production, and better batch-to-batch reproducibility [14,15,16]. Furthermore, small molecule organic materials could exhibit higher charge carrier mobility, as well as tuning more easily their band structure to absorb sunlight efficiently [17]. These advantages make small molecule OSCs strong competitors to polymer solar cells. Small molecule organic materials are well on their way to outperform polymers in OSC applications [18,19]. Therefore, it is believed that small-molecule OSCs are promising to realize their commercial application in the future. However, the PCEs of small-molecule OSCs still lagged behind that of the polymer counterparts [20,21,22]. Therefore, it is still a significant challenge for the research community to develop novel, high-performance, and desirable donor materials to improve the PCEs of small molecule OSCs. In order to maximize small molecule OSC performance, the energy of the frontier molecular orbitals (FMO) including the HOMO and LUMO of the designed and synthesized donor material should be suitable to those of [6,6]-phenyl-C_61_-butyric acid methyl ester (PCBM) and its derivatives [23,24], an excellent acceptor of OSCs [25]. Furthermore, donating material should exhibit a broad absorption region, high and balanced charge transfer properties, and high ambient stability. A number of studies demonstrated some guidelines to experiments, where useful insights have been provided to help understand the nature of the molecules [26,27,28,29].

It is well known that there are three major parameters that determine the PCEs of OSCs: Open circuit voltage (*V*_oc_), short circuit current (*J*_sc_), and fill factor (*FF*) [30]. In order to realize highly-efficient small molecule-based OSCs, an ideal donor material should have a low energy gap and deep highest-occupied molecular orbital energy level. A low energy gap is beneficial for effective optical absorption, providing a larger *J*_sc_, while a deep highest-occupied molecular orbital energy level can increase the *V*_oc_. In addition, the high hole mobility is also crucial for the carrier transport to improve the *J*_sc_ and *FF* [31]. In OSC research, the push-pull organic compounds, containing π-center with electron donors and acceptors on the terminal sites of the conjugated backbone, are regarded as one of the most promising molecular materials [12,13,18]. This type of molecular structure can enhance intramolecular charge transfer (ICT), yielding higher molar absorptivity. At the same time, they can lower the material band gap and extend the absorption spectrum towards longer wavelengths. Additionally, the electronic energy levels and band gaps can be tuned effectively through adjusting the acceptor and donor units, and π-bridge units or length [32,33]. Among the various push-pull organic compounds investigated, diketopyrrolopyrrole (DPP)-based molecules have been regarded as a promising core building block for small molecule OSC materials due to their strong light absorption, good photochemical stability, excellent charge carrier mobility, and easy synthesis [34,35,36,37]. Recently, some DPP-based small molecule OSC materials have been reported [38]. It was found that these molecules have good charge transport, film morphology, and optical properties.

**Scheme 1:**
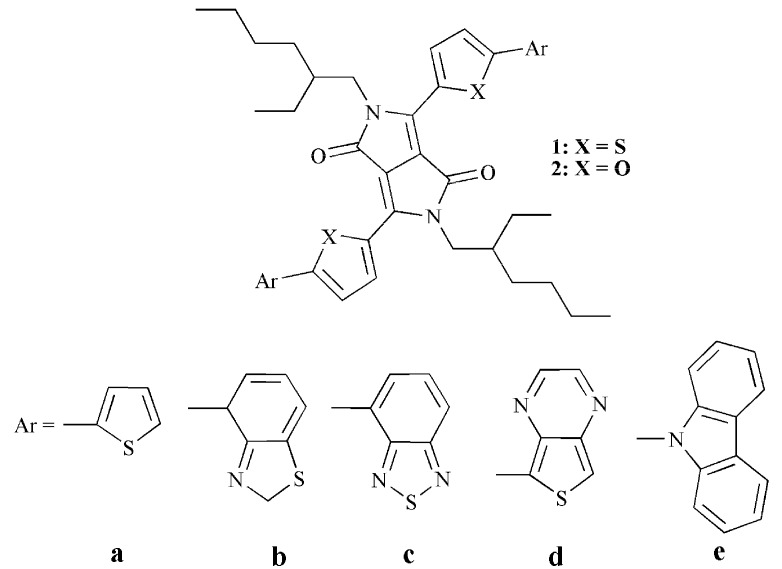
Molecular structures of the investigated molecules.

With the above considerations, in this work, we report the investigation of both optical and charge transporting properties from a theoretical point of view for DPP-based small molecules. We designed a series of DPP-based small molecules with DPP derivative 2,5-bis(2-ethylhexyl)pyrrolo[3,4-c]pyrrole-1,4(2H,5H)-dione (**BEDPP)** as core, thiophene or furan as conjugate π-bridges (**CB**), and aromatic derivatives (**Ar**) as end groups for OSCs applications (Scheme 1). The purpose of this molecular architecture was to investigate the relationship between topologic structure and optical, as well as electronic, properties to provide a demonstration for the rational design of a novel candidate for small molecule OSC materials. We have also predicted the mobility of the designed molecules.

## 2. Results and Discussion

### 2.1. Frontier Molecular Orbitals

**Figure 1:**
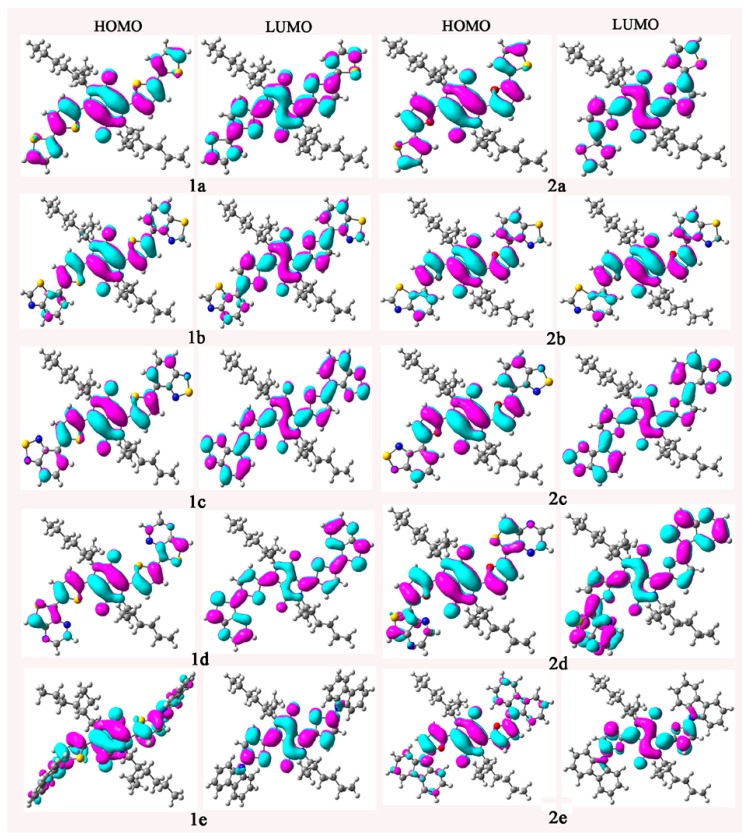
The electronic density contours of the frontier orbitals for the compounds under investigation at the B3LYP/6-31G(d,p) level.

To characterize the optical and electronic properties, it is useful to examine the FMOs of the compounds under investigation. The qualitative molecular orbital representations of the HOMOs and LUMOs of the compounds under investigation are plotted in Figure 1. The total and partial densities of states (TDOS and PDOS) on each fragment of the investigated molecules around the HOMO-LUMO gaps were calculated based on the current level of theory. The HOMOs and LUMOs contributions of individual fragments (in%) to the FMOs of the investigated molecules are given in Table 1. The FMOs of the compounds under investigation show π characteristics as visualized in Figure 1. The distribution patterns of FMOs are spread over the whole molecules for the investigated molecules. It implies that the spatial overlap between the HOMOs and LUMOs are strong, which may result in stronger optical absorption corresponding to the transition from HOMOs to LUMOs. The HOMOs of the investigated molecules are mainly localized on the **BEDPP** and **CB** fragments with only minor contributions from **Ar** fragments. The sum contributions of **BEDPP** and **CB** fragments of HOMOs are larger than 80%, while the corresponding contributions of **Ar** fragments are within 20%, respectively. Similar phenomena are found for the LUMOs of **1a**, **1b**, **1e**, **2a**, **2b**, and **2e**. The LUMOs mainly reside at the **BEDPP** and **CB** fragments with only minor contributions from **Ar** fragments. The sum contributions of **BEDPP** and **CB** fragments of LUMOs are larger than 84.6%, while the corresponding contributions of **Ar** fragments are within 15.4%, respectively. However, for the LUMOs of **1c**, **1d**, **2c**, and **2d**, the sum contributions of **BEDPP** and **CB** fragments are almost equal to those of **Ar** fragments. These results reveal that the different π-bridge units and end groups have obvious effects on the distribution of FMOs for the compounds under investigation. The distribution patterns of the FMOs of the compounds under investigation provide a remarkable signature for the intramolecular charge transfer (ICT) character of the vertical S_0_ → S_1_ transition. Analysis of the FMOs indicates that the excitation of the electron from the HOMOs to LUMOs leads the electronic density to flow mainly from the **BDDPP** fragments to **CB** and **Ar** fragments for **1a**, **1b**, and **2b**. The percentages of charge transfer are the differences between the contributions of fragments for LUMOs and the corresponding contributions for HOMOs in the compounds under investigation. The percentages of charge transfer from **BEDPP** fragments to **CB** and **Ar** fragments for **1a**, **1b**, and **2b** are 8.5%, 12.8%, and 5.2%, respectively. For **1c**, **1d**, **2c**, and **2d**, the excitation of the electron from the HOMOs to LUMOs leads the electronic density to flow mainly from **BEDPP** and **CB** fragments to **Ar** fragments, while the corresponding electronic density flow mainly from **BEDPP** and **Ar** fragments to **CB** fragments for **2a**. However, for **1e** and **2e**, the excitation of the electron from the HOMOs to LUMOs leads the electronic density to flow mainly from **Ar** fragments to **BEDPP** and **CB** fragments, the percentages of charge transfer from **Ar** fragments to **BEDPP** and **CB** fragments are about 12.1% and 13.6%, respectively. The results displayed in Table 1 reveal that the **BEDPP** fragments serve as donors and **CB** and **Ar** fragments serve as acceptors for **1a**, **1b**, and **2b**, while the **BEDPP** and **CB** fragments serve as donors and **Ar** fragments serve as acceptors for **1c**, **1d**, **2c**, and **2d**. The **BEDPP** and **Ar** fragments serve as donors and **CB** fragments serve as acceptor for **2a**. However, the **Ar** fragments serve as donors and **BEDPP** and **CB** fragments serve as acceptors for **1e** and **2e**.

**Table 1:** The HOMOs and LUMOs contributions of individual fragments (in%) to the FMOs of the investigated molecules at the B3LYP/6-31G(d,p) level.

Species	HOMOs			LUMOs		
	BEDPP ^a^	CB ^b^	Ar ^c^	BEDPP ^a^	CB ^b^	Ar ^c^
**1a**	55.3	30.2	14.5	46.8	37.8	15.4
**1b**	61.1	29.2	9.7	48.3	37.5	14.2
**1c**	58.7	29.0	12.4	28.0	25.3	46.8
**1d**	52.1	28.5	19.4	23.4	23.3	53.3
**1e**	55.2	28.2	16.6	55.3	40.2	4.50
**2a**	54.4	30.7	14.9	52.6	33.1	14.3
**2b**	57.9	30.2	11.9	52.7	32.7	14.6
**2c**	56.6	29.8	13.6	31.0	21.1	47.9
**2d**	54.6	27.7	17.7	26.4	19.3	54.4
**2e**	51.4	29.2	19.4	60.0	34.2	5.80

^a^ BEDPP: 2,5-bis(2-ethylhexyl)pyrrolo[3,4-c]pyrrole-1,4(2H,5H)-dione moieties; ^b^ CB: conjugate bridge moieties; ^c^ Ar: aromatic moieties.

**Figure 2:**
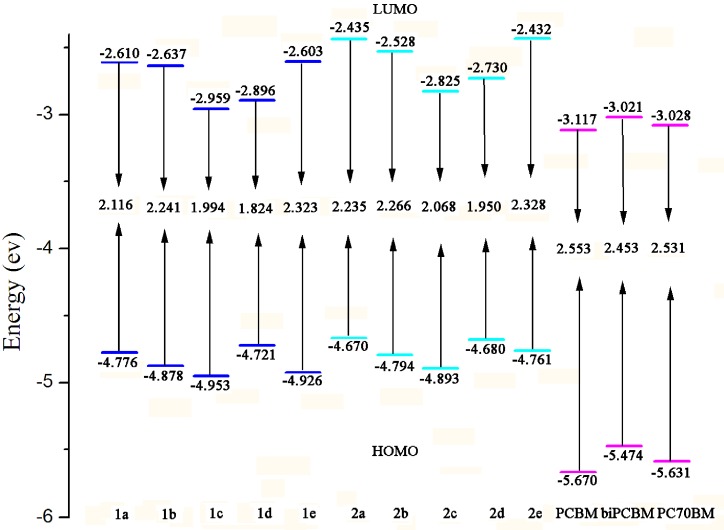
Evaluation of calculated FMO energies for investigated molecules as well as FMO energies for PCBM, bisPCBM, and PC70BM at the B3LYP/6-31G(d,p) level.

Another way to understand the influence of the optical and electronic properties is to analyze the *E*_HOMO_, *E*_LUMO_, and *E*_g_. The *E*_HOMO_, *E*_LUMO_, and *E*_g_ of the designed molecules, PCBM and its derivatives bisPCBM and PC70BM were calculated and the results are given in Figure 2. As shown in Figure 2, for molecules with thiophene π-bridge (**1a**–**1e**), the *E*_HOMO_ values are in the order of **1d** > **1a** > **1b** > **1e** > **1c** and the sequence of *E*_LUMO_ values is **1e** > **1a** > **1b** > **1d** > **1c**. Therefore, the *E*_g_ values are in the order of **1e** > **1b** > **1a** > **1c** > **1d**. This shows that molecules with benzo[d]thiazole (BT), benzo[c][1,2,5]thiadiazole (BTD), and 9H-carbazole (CZ) end groups possess lower *E*_HOMO_, while a molecule with a thieno[3,4-b]pyrazine (TP) end group has higher *E*_HOMO_ compared with molecules with thiophene end groups. For *E*_LUMO_, molecules with BT, TP, and BTD end groups possess lower *E*_LUMO_, while molecules with CZ end groups has higher *E*_LUMO_ in comparison with molecules with thiophene end groups. The *E*_g_ value of molecules with BT and CZ end groups are larger, while the corresponding values of molecules with TP and BTD end groups are smaller than that of molecules with thiophene end groups. For molecules with furan π-bridge (**2a**–**2e**), the *E*_HOMO_ values are in the order of **2a** > **2d** > **2e** > **2b** > **2c** and the sequence of *E*_LUMO_ values is **2a** > **2e** > **2b** > **2d** > **2c**. Thus, the *E*_g_ values are in the order of **2e** > **2b** > **2a** > **2c** > **2d**. This indicates that molecules with BT, BTD, TP, and CZ end groups possess lower both *E*_HOMO_ and *E*_LUMO_ values compared with molecules with thiophene end groups. The BT and CZ end groups increase, while the TP and BTD end groups decrease the *E*_g_ values compared with that of molecules with thiophene end groups. Furthermore, compared the *E*_HOMO_, *E*_LUMO_, and *E*_g_ of molecules with thiophene π-bridges with those with furan π-bridges, one can find that the *E*_HOMO_, *E*_LUMO_, and *E*_g_ values of with furan π-bridges are larger than those of molecules with thiophene π-bridges. These results suggest that the different π-bridges and end groups have effects on the *E*_HOMO_, *E*_LUMO_, and *E*_g_ for the compounds under investigation.

It is well-known that PCBM, bisPCBM, and PC70BM are excellent acceptors for organic solar cells [39,40,41]. Therefore, we choose these three fullerene derivatives as acceptors in our work. As shown in Figure 2, the *E*_LUMO_ values of the designed molecules are higher than those of PCBM, bisPCBM, and PC70BM, respectively. The differences between the *E*_HOMO_ of the designed molecules and the *E*_LUMO_ of PCBM are 1.563~1.836 eV, while the corresponding values of bisPCBM and PC70BM are larger than 1.649 and 1.642 eV, respectively. These results imply that the designed molecules can provide better matches of FMOs to PCBM, bisPCBM, and PC70BM. Therefore, different π-bridges and aromatic end groups can tune the FMOs of derivatives more suitable to PCBM, bisPCBM, and PC70BM.

### 2.2. Absorption Spectra

Table 2 presents the absorption region *R*, the longest wavelength (λ_abs_) of absorption spectrum, the oscillator strength (*f*), and main configurations of the designed molecules. The absorption wavelengths λ_abs_ and the oscillator strength *f* of the first fifteen excited states for the compounds under investigation are listed in Tables S1 and S2 in the Supporting Information. The λ_abs_ value of **1a** is in agreement with the experimental result [38], the deviation is 9 nm. This reveals that the level of theory we selected is reasonable for this type of system. The absorptions of the compounds under investigation are assigned to the S_0_ → S_1_ electronic transitions and HOMOs → LUMOs excitations play a dominant role. From Table 2, one can find that the λ_abs_ of **1c**, **1d**, **2c**, and **2d** have bathochromic shifts, while the corresponding λ_abs_ values of **1b**, **1e**, **2a**, **2b**, and **2e** have hypsochromic shifts compared with that of the parent compound **1a**. The λ_abs_ values are in the order of **nd** > **nc** > **na** > **nb** > **ne** (*n* = 1 and 2), which is in excellent agreement with the corresponding reverse order of *E*_g_ values displayed in Figure 2. It indicates that the introduction of BT and CZ end groups decrease, while the introduction of TP and BTD end groups increase the λ_abs_ values compared with molecules with thiophene end groups for designed molecules. For **1a**–**1e**, the sequence of their *R* values is **1d** > **1c** > **1a** > **1b** > **1e**. It suggests that the introduction of BT and CZ end groups results in smaller *R* values, while the introduction of TP and BTD end groups lead to the increase of *R* values compared with molecules with thiophene end groups. However, for **2a**–**2e**, their *R* values are in the order of **2d** > **2c** > **2b** > **2e** > **2a**. It indicates that the introduction of BT, CZ, TP, and BTD end groups leads to the increase of *R* values compared with the thiophene end group. The oscillator strength for an electronic transition is proportional to the transition moment [42]. In general, larger oscillator strength corresponds to larger experimental absorption coefficient. The order of the predicted *f* values are in the decreasing order of **nb** > **na** > **ne** > **nc** > **nd** (*n* = 1 and 2). This indicates that the introduction of BT end group increases, while the introduction of BTD, TP, and CZ end groups slightly decreases the *f* values compared with thiophene end groups for the designed molecules. Furthermore, a careful inspection of the results displayed in Table 2 reveals clearly that the λ_abs_ and *R* values of molecules with BTD and TP end groups are larger than those of other molecules. It suggests that molecules with BTD and TP end groups can lower the material band gap and extend the absorption spectrum towards longer wavelengths. The λ_abs_ and *R* values of molecules with BT and CZ end groups are smaller slightly than those of parent molecule **1a**. Therefore, molecules under investigation own the large λ_abs_, *f*, and *R* values. The designed molecules could be used as solar cell material with intense, broad absorption spectra.

**Table 2:** Predicted absorption region *R*, the longest wavelength of absorption, corresponding oscillator strength *f*, and main configurations of the compounds under investigation at the TD-B3LYP/6-31G(d,p)//B3LYP/6-31G(d,p) level.

Species	λ_abs_ (nm)	*f*	Main configurations	*R* (nm) ^a^
**1a**	599	0.97	H → L (0.71)	295
**1b**	588	0.98	H → L (0.71)	281
**1c**	684	0.82	H → L (0.70)	340
**1d**	746	0.80	H → L (0.70)	387
**1e**	575	0.87	H → L (0.70)	261
**2a**	583	0.74	H → L (0.71)	209
**2b**	581	0.81	H → L (0.71)	288
**2c**	669	0.61	H → L (0.70)	354
**2d**	716	0.56	H → L (0.70)	357
**2e**	574	0.73	H → L (0.70)	271
Exp ^b^	590			

^a^ *R* denotes for the difference of the longest and shortest wavelength values with oscillator strength larger than 0.01 considering the first fifteen excited states; ^b^ Experimental data for **1a** were taken from Ref. [38].

### 2.3. Reorganization Energies

Understanding the relationship between molecular structure and charge transport property of the material is a key factor for designing good candidates for solar cell devices. It is well-known that the lower the reorganization energy values, the higher the charge transfer rate [43,44]. The calculated reorganization energies for hole and electron are listed in Table 3. The results displayed in Table 3 show that the calculated λ_h_ values of **1c**, **1d**, **2c**, and **2d** are smaller than that of *N*,*N*′-diphenyl-*N*,*N*′-bis(3-methlphenyl)-(1,1′-biphenyl)-4,4′-diamine (TPD), which is a typical hole transport material (λ_h_ = 0.290 eV) [45]. The calculated λ_h_ values of **1a**, **1b**, **2a**, and **2b** are slightly larger than that of TPD. This implies that the hole transfer rates of **1c**, **1d**, **2c**, and **2d** might be higher than that of TPD, while the hole transfer rates of **1a**, **1b**, **2a**, and **2b** are almost equal to that of TPD. The λ_h_ values of **1e** and **2e** are larger than that of TPD. It suggests that their hole transfer rates might be lower than that of TPD. The λ_e_ values of the designed molecules except for **1e** are smaller than that of tris(8-hydroxyquinolinato)aluminum(III) (Alq3), which is a typical electron transport material (λ_e_ = 0.276 eV) [46], indicating that the electron transfer rates of the designed molecules except for **1e** might be higher than that of Alq3. For molecules with thiophene π-bridges (**1a**–**1e**), both the λ_h_ and λ_e_ value of **1d** and **1c** are smaller, while the corresponding values of **1b** and **1e** are larger than those of **1a**. This suggests that the introduction of TP and BTD end groups increases, while the introduction of BT and CZ end groups decreases the electron and hole transfer rates compared with molecules with thiophene end groups. For molecules with furan π-bridges (**2a**–**2e**), the λ_h_ values of **2b**, **2d**, and **2c** are smaller, while the corresponding value of **2e** is larger than that of **2a**. On the contrary, the λ_e_ values of **2b**, **2d**, and **2e** are larger, while the corresponding value of **2c** is smaller than that of **2a**. This indicates that the introduction of BT, BTD, and TP end groups increases, while the introduction of CZ end group decreases the hole transfer rates compared with thiophene end groups. However, BT, TP, and CZ end groups decrease, while BTD end groups increase the electron transfer rates compared with thiophene end groups. From Table 3, one can find that **ne** (*n* = 1 and 2) have the largest λ_h_ and λ_e_ values, while **nc** (*n* = 1 and 2) own the smallest λ_h_ and λ_e_ values, respectively. Inspection of the results displayed in Table 3 reveals that the designed molecules can be used as promising hole transport materials except for molecules with CZ end groups. The designed molecules can be used as promising electron transport materials from the stand point of the smaller reorganization energy except for molecules with thiophene π-bridges and CZ end groups.

**Table 3:** Calculated λ_e_ and λ_h_ (both in eV) of the compounds under investigation at the B3LYP/6-31G(d,p) level.

Species	λ_h_	λ_e_
**1a**	0.293	0.160
**1b**	0.295	0.199
**1c**	0.266	0.154
**1d**	0.258	0.171
**1e**	0.489	0.285
**2a**	0.301	0.153
**2b**	0.291	0.159
**2c**	0.273	0.143
**2d**	0.287	0.191
**2e**	0.426	0.210

### 2.4. Calculated Crystal Structure and Transport Properties

**Figure 3:**
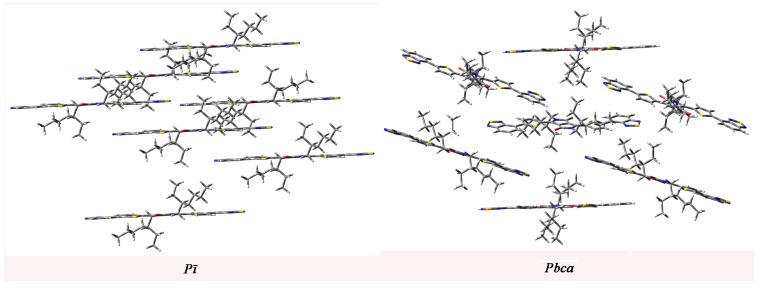
Herringbone structures of **1c** in different space groups.

**Figure 4:**
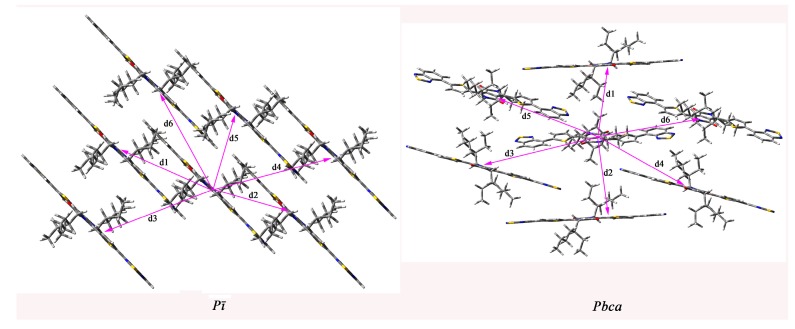
Crystal structures and hopping routes of **1c** in different space groups.

We calculated the mobility of the designed molecules to study their charge transport property. The total energies of the predicted crystal structures for the designed molecules in different space groups are summarized in Tables S3 and S4 in the Supporting Information. The lattice constants of the designed molecules with the lowest total energies are listed in Table S5 in the Supporting Information. The transmission paths are selected according to the optimized crystal structures. We arbitrarily choose one molecule in the crystal as the carrier donor and take all its neighboring molecules as paired elements. Each pair is defined as a transmission path. Then the charge transfer integral can be calculated according to the transmission path. The mobility can be estimated from the Einstein relation. We predict the mobility of the designed molecules with the lowest total energies. In order to understand the crystal structures effect on the charge transfer integral and mobility, we select **1c** as representative of the system under investigation. The predicted crystal structures of **1c** with two lowest total energies belong to space groups *P*ī and *Pbca* and shown in Figure 3. We predict the charge transfer integral and mobility of **1c** in these two space groups. The most important pathways (dimers) in space groups *P*ī and *Pbca* are shown in Figure 4. Then the charge transfer integral can be calculated according to the transmission path, and the mobility can be estimated from the Einstein relation. The calculated transfer integrals of **1c** for holes and electrons in space groups *P*ī and *Pbca* are listed in Table 4. The calculated transfer integrals of others designed molecules with the lowest total energies for holes and electrons are listed in Table S6 in the Supporting Information. The data in Table 4 demonstrate that the electronic coupling is determined by the relative distance and orientations of the interacting molecules [47]. Furthermore, **1c** possesses the largest absolute electron and hole coupling values in pathways 1, 3, and 5 for space group *P*ī and in pathways 3, 4, and 5 for space group *Pbca*. It reveals that the orientation of the interacting molecules is the key factor of hole or electron coupling for **1c**, because the co-facial stacking structure is expected to provide more efficient orbital overlap leading to the most efficient charge transfer route [47]. The calculated electron and hole mobility of the compounds under investigation are listed in the Table 5. The values of hole mobility of **1c** for *P*ī and *Pbca* space group (2.00 × 10^−3^ and 2.14 × 10^−3^ cm^2^/Vs) are larger than that of TPD (1.0 × 10^−3^ cm^2^/Vs) [48], respectively. The values of electron mobility of **1c** for *P*ī and *Pbca* space group are 8.97 × 10^−2^ and 5.11 × 10^−2^ cm^2^/Vs, respectively. The values of electron mobility for *P*ī and *Pbca* space group are larger than that of hole mobility, respectively. It shows that different space groups lead to different mobility values; that is to say, the stacking structure is the most important factor for the molecular mobility property. The theoretical prediction shows that **1c** can be made as hole and electron transfer materials used for solar cells. Moreover, it also has balanced charge transport characteristics, which agrees with the result of reorganization energy.

The results displayed in Table 5 show that the hole mobility values of **1a**, **1c**, **1d**, **2a**, and **2b** are larger, while the corresponding values of **1b**, **1e**, and **2c**–**2e** are smaller than that of **TPD**. Furthermore, from Table 5, one can find that the hole mobility values are in the orders of **1d** > **1a** > **1c** > **1b** > **1e** and **2b** > **2a** > **2d** > **2e** > **2c** for **1a**–**1e** and **2a**–**2e**, respectively. It suggests that the introduction of the TP end group can increase, while the introduction of BT, BTD, and CZ end groups leads to the decrease for the hole mobility values compared with molecule with thiophene end groups for **1a**–**1e**. The introduction of the BT end group increases, while the introduction of BTD, TP, and CZ end groups decreases the hole mobility values compared with molecule with thiophene end groups for **2a**–**2e**. The sequences of electron mobility are **1d** > **1c** > **1a** > **1b**> **1e** and **2a** > **2c** > **2b** > **2d**> **2e** for **1a**–**1e** and **2a**–**2e**, respectively. This clearly shows that the electron mobility values can be decreased by introduction of BT and CZ end groups, while introduction of BTD and TP end groups increases the electron mobility values compared with molecule with thiophene end groups for **1a**–**1e**. The introduction of BT, BTD, TP, and CZ groups leads to the decrease for the electron mobility values compared with molecule with thiophene end groups for **2a**–**2e**. Inspection of the results displayed in Table 5 reveals clearly that the electron mobility values of **1a**–**1d**, **2a**, **2c**–**2e** are larger than those of hole mobility values, respectively. However, the electron mobility value of **2b** is smaller than that of hole mobility value. The electron mobility value of **1e** is almost equal to that of hole mobility value. Considering the reorganization energy for designed molecules above, the compounds under investigation can be made as electron transfer materials except for **1e**, particularly for **1a** and **1c**. The **1a**, **1c**, **1d**, **2a**, and **2b** can be made as hole transfer materials. This suggests that the compounds under investigation can be made as hole and/or electron transfer materials used for solar cells.

**Table 4:** The center-center distance and the corresponding hole and electron coupling between the dimer in all of the nearest neighbor pathways for **1c** in different space groups.

Space Groups	Pathway	Distance (Å)	Electron Coupling (eV)	Hole Coupling (eV)
*P*ī	1	13.450	−9.00 × 10^−3^	−3.22 × 10^−4^
	2	13.450	−9.00 × 10^−3^	−3.22× 10^−4^
	3	16.936	−3.28 × 10^−7^	1.25 × 10^−6^
	4	16.936	−3.28 × 10^−7^	1.25 × 10^−6^
	5	11.223	1.50 × 10^−3^	−3.20 × 10^−3^
	6	13.509	1.50 × 10^−8^	1.04 × 10^−8^
*Pbca*	1	13.373	−5.86 × 10^−6^	8.19 × 10^−6^
	2	13.373	−5.86 × 10^−6^	8.19 × 10^−6^
	3	14.824	−5.80 × 10^−3^	2.50 × 10^−3^
	4	14.824	−5.80 × 10^−3^	2.50 × 10^−3^
	5	14.824	−5.80 × 10^−3^	2.50 × 10^−3^
	6	15.591	−6.50 × 10^−3^	5.79 × 10^−4^

**Table 5:** The electron and hole mobility of the designed molecules. [T = 298 K, in cm^2^/Vs].

Species	Space Groups	Electron Mobility	Hole Mobility
**1a**	*P2_1_2_1_2_1_*	1.05 × 10^−^^2^	8.18 × 10^−^^3^
**1b**	*Pna21*	2.21 × 10^−^^4^	1.18 × 10^−^^4^
**1c**	*P*ī	8.97 × 10^−2^	2.00 × 10^−3^
**1c**	*Pbca*	5.11 × 10^−2^	2.14 × 10^−3^
**1d**	*C2/c*	0.136	9.57 × 10^−^^3^
**1e**	*P2_1_/c*	2.45 × 10^−^^6^	7.75 × 10^−^^6^
**2a**	*P2_1_*	7.561 × 10^−^^3^	1.77 × 10^−^^3^
**2b**	*P2_1_*	4.22 × 10^−^^5^	7.13 × 10^−^^2^
**2c**	*Cc*	8.18 × 10^−^^4^	1.31 × 10^−^^5^
**2d**	*P2_1_2_1_2_1_*	3.14 × 10^−^^5^	2.56 × 10^−^^5^
**2e**	*P*ī	2.55 × 10^−^^5^	1.78 × 10^−^^5^

## 3. Computational Methods

All calculations have been carried out using Gaussian 09 package [49]. The equilibrium structures of the compounds under investigation, including neutral, cationic, and anionic molecules, were optimized using the B3LYP functional with the 6-31G(d,p) basis set. The harmonic vibrational frequency calculations using the same methods as for the geometry optimizations were used to ascertain the p3resence of a local minimum. To compare the energies of the FMOs for donors and acceptors, the electronic properties of PCBM and its derivatives were calculated at the B3LYP/6-31G(d,p) level based on the optimized structures at the B3LYP/6-31G(d) level. The absorption spectra of the compounds under investigation were predicted using the TD-B3LYP/6-31G(d,p) method based on the optimized geometries.

According to Marcus theory [43,44], the charge transport can be considered as a hopping process in the organic solid. The charge transfer rate can be represented by means of the following equation:

(1) K=(V2ℏ)(πλkBT)12exp(−λ4kBT)

where *T* represents the absolute temperature, *k*_B_ is the Boltzmann constant, λ and *V* correspond to the reorganization energy and transfer integral, respectively. Inspection of Equation (1) clearly reveals that the reorganization energy and transfer integral play a dominant role in determining the charge transfer rate. The minimal reorganization energy and the maximal transfer integral can lead to the increase of charger transfer rates. For the reorganization energies λ, they consist of external reorganization energy (λ_ext_) and internal reorganization energy (λ_int_). λ_ext_ represents the effect of polarized medium on charge transfer. The internal reorganization is due to the change between ionic and neutral states. The predicted λ_ext_ values in pure organic condensed phases are much smaller than their λ_int_ counterparts and can be neglected [50,51,52]. Moreover, there is a clear correlation between λ_int_ and charge transfer rate in literature [53]. Therefore, we focus on the λ_int_ of the isolated active organic π-conjugated systems exclusively. The reorganization energies for electron (λ_e_) and hole (λ_h_) can be calculated by equations [54]:

(2) $$\lambda e=\left(E_{0}^{-}-E_{-}^{-}\right)+\left(E_{-}^{0}-E_{0}^{0}\right)$$

(3) $$\lambda h=\left(E_{0}^{+}-E_{+}^{+}\right)+\left(E_{+}^{0}-E_{0}^{0}\right)$$

where $E_{0}^{+}$($E_{0}^{-}$) is the energy of the cation (anion) calculated with the optimized structure of the neutral molecule. Similarly, $E_{+}^{+}$($E_{-}^{-}$) is the energy of the cation (anion) calculated with the optimized cation (anion) structure, $E_{+}^{0}$($E_{-}^{0}$) is the energy of the neutral molecule calculated at the cationic (anionic) state, and $E_{0}^{0}$ is the energy of the neutral molecule in ground state. For comparing with the interested results reported previously [45,46], the λ_e_ and λ_h_ of the molecules were calculated at the B3LYP/6-31G(d,p) level on the basis of the single point energy.

For the charge transfer integral of hole (electron) transfer, they can be predicted using equation [55,56]:

(4) $$V_{ij}=\langle \varphi_{1}^{0}|{\hat{F}}^{0}|\varphi_{2}^{0}\rangle$$

*V*_ij_ is the transfer integral. $\varphi_{1}^{0}$ and $\varphi_{2}^{0}$ represent the unperturbed frontier orbital of molecules 1 and 2 in the dimer, respectively. ${\hat{F}}^{0}$ is the Kohn-Sham-Fock operator of the dimer obtained with the unperturbed density matrix, which can be evaluated by the molecular orbitals and density matrix of the two individual molecules. The pw91pw91/6-31G(d) method is employed to calculate the transfer integral. This method gave a reasonable description for intermolecular coupling term previously [57]. The molecular crystal structure is predicted by the module Polymorph of the software package Materials Studio [58]. The geometry of the cluster models used in the present study was taken from the B3LYP/6-31G(d,p) level.

The drift mobility of hopping μ, can be evaluated from the Einstein equation:

(5) $$\mu =\frac{e}{k_{B}T}D$$

where *e* is the electronic charge, *D* is the diffusion coefficient, which can be evaluated as [59]:

(6) $$D=\underset{t\to \infty }{\lim}\frac{1}{2n}\frac{\langle x{\left(t\right)}^{2}\rangle }{t}\approx \frac{1}{2n}\sum_{i}d_{i}^{2}k_{i}p_{i}=\frac{1}{2n}\frac{\sum_{i}d_{i}^{2}k_{i}^{2}}{\sum_{i}k_{i}}$$

Here *d*_i_ is the center mass distance to neighbor *i*, *n* is the spatial dimension of the crystal, and *k*_i_ is the hopping rate due to charge transfer to *i*th neighbor. *P*_i_ represents the relative probability for charge transfer to a particular *i*th neighbor.

(7) $$p_{i}=\frac{k_{i}}{\sum_{j}k_{i}}$$

## 4. Conclusions

In the present work, we investigated a series of DPP-based small molecules with DPP derivative 2,5-bis(2-ethylhexyl)pyrrolo[3,4-c]pyrrole-1,4(2H,5H)-dione (**BEDPP)** as core, thiophene or furan as conjugate π-bridges (**CB**), and aromatic derivatives (**Ar**) as end groups for OSC applications. The FMO analyses have turned out that the molecules can lower the material band gap and extend the absorption spectrum towards longer wavelengths. Our results reveal that the molecules under investigation own the longest wavelength of absorption spectra, oscillator strength, and absorption region values. They could be used as solar cell material with intense broad absorption spectra. Our results suggest that the optical, electronic, and charge transport properties are affected by the introduction of different end groups. The mobility values of the designed molecules are also investigated. Our results show that the designed molecules are expected to be promising candidates for electron and/or hole transport materials for solar cells. On the basis of the investigated results, we suggest that molecules under investigation are suitable donors for PCBM, bisPCBM, and PC70BM as acceptors of solar cells.

## Supplementary Materials

Supplementary materials can be found at http://www.mdpi.com/1422-0067/16/09/20326/s1.
