# Supplementary material for: Rational Design of Diketopyrrolopyrrole-Based Small Molecules as Donating Materials for Organic Solar Cells
Source: Int J Mol Sci. 2015 Aug 27;16(9):20326–43. doi: 10.3390/ijms160920326 (PMC4613206; doi:10.3390/ijms160920326)
Supplement: Supplementary file 1 [file ijms-16-20326-s001.pdf]

Supplementary Information

Table S1. The λ_{abs} and corresponding oscillator strength *f* (in parenthesis) of the first fifteen excited states for **1a**–**1e** obtained by the TD−B3LYP/6−31G(d,p) level.

Species	1a	1b	1c	1d	1e
S_1	599(0.97)	588(0.98)	684(0.82)	746(0.80)	575(0.87)
S_2	452(0.00)	439(0.01)	604(0.00)	659(0.00)	479(0.00)
S_3	410(0.30)	400(0.16)	504(0.27)	522(0.34)	453(0.07)
S_4	401(0.00)	395(0.15)	438(0.00)	479(0.00)	426(0.00)
S_5	386(0.00)	387(0.02)	425(0.39)	464(0.26)	426(0.00)
S_6	368(0.00)	371(0.11)	409(0.00)	434(0.01)	390(0.00)
S_7	349(0.25)	370(0.05)	393(0.02)	414(0.00)	376(0.00)
S_8	337(0.00)	351(0.002)	381(0.00)	409(0.00)	361(0.02)
S_9	320(0.00)	341(0.00)	380(0.00)	404(0.00)	337(0.04)
S_{10}	319(0.10)	332(0.01)	373(0.00)	403(0.00)	337(0.00)
S_{11}	313(0.34)	329(0.02)	354(0.00)	387(0.00)	331(0.00)
S_{12}	308(0.00)	325(0.04)	351(0.00)	385(0.00)	326(0.30)
S_{13}	304(0.02)	316(0.02)	345(0.04)	374(0.00)	319(0.06)
S_{14}	303(0.00)	313(0.17)	344(0.03)	359(0.08)	316(0.00)
S_{15}	398(0.00)	307(0.23)	339(0.00)	356(0.00)	314(0.01)

Table S2. The λ_{abs} and corresponding oscillator strength *f* (in parenthesis) of the first fifteen excited states for **2a**–**2e** obtained by the TD−B3LYP/6−31G(d,p) level.

Species	2a	2b	2c	2d	2e
S_1	583(0.74)	581(0.81)	669(0.61)	716(0.56)	574(0.73)
S_2	433(0.00)	430(0.00)	598(0.00)	640(0.00)	454(0.00)
S_3	403(0.25)	400(0.22)	504(0.27)	518(0.33)	419(0.18)
S_4	390(0.00)	393(0.02)	430(0.00)	462(0.03)	415(0.00)
S_5	376(0.00)	384(0.05)	415(0.37)	438(0.25)	415(0.00)
S_6	364(0.00)	369(0.23)	398(0.00)	420(0.02)	381(0.00)
S_7	345(0.38)	364(0.02)	389(0.02)	405(0.00)	357(0.00)
S_8	331(0.00)	344(0.00)	376(0.01)	378(0.15)	357(0.00)
S_9	309(0.00)	335(0.01)	372(0.00)	402(0.01)	347(0.04)
S_{10}	307(0.02)	332(0.00)	361(0.00)	388(0.00)	346(0.00)
S_{11}	307(0.00)	329(0.03)	349(0.00)	386(0.00)	324(0.00)
S_{12}	398(0.00)	317(0.11)	346(0.10)	378(0.01)	318(0.28)
S_{13}	389(0.00)	303(0.25)	340(0.00)	361(0.06)	311(0.00)
S_{14}	374(0.04)	298(0.14)	329 (0.00)	359(0.04)	303(0.10)
S_{15}	273(0.00)	293(0.02)	315(0.02)	352(0.00)	298(0.00)

Table S3. Calculated total energies of **1a**–**1e** in different space groups.

Space Groups	1a	1b	1c	1d	1e
C2	267.10664755	321.45638233	225.97183574	376.16415328	879.83611392
C2/c	269.88335381	332.26630469	228.28860482	373.15880464	868.31983476
Cc	260.14853111	324.03331709	227.39384311	373.72921396	879.48598871
$P2_1$	269.71169906	318.77881185	220.98170266	374.26251003	871.26386752
$P2_1/c$	264.46285211	328.58530947	226.52206532	374.16464004	865.94238578
$P2_12_12_1$	258.62516273	321.33589767	226.24788602	374.98938481	880.81192845
$P\bar{1}$	263.27380154	321.98033124	219.08346663	375.30463151	869.53027788
Pbca	265.48190996	330.26320365	219.22909939	380.68435027	879.09792126
Pbcn	269.61362945	321.25929840	228.08771363	381.18545364	879.77181353
$Pna2_1$	266.18533080	315.27651208	222.44344036	374.93555255	876.19982055

Table S4. Calculated total energies of **2a**–**2e** in different space groups.

Space Groups	2a	2b	2c	2d	2e
C2	236.53285448	287.22982116	198.96449301	324.87622013	817.08018345
C2/c	239.46768323	294.80879220	204.80305955	334.28605557	815.80933012
Cc	235.32105115	294.94393820	191.17289639	338.24251903	810.63411494
$P2_1$	230.23637381	275.64353002	198.65310594	324.59587230	808.35244122
$P2_1/c$	232.72488878	293.13462821	193.32358123	331.00351998	811.57894187
$P2_12_12_1$	240.80366316	294.50413578	197.37331724	323.30451552	809.85143134
$P\bar{1}$	239.54100556	290.71834608	195.65673953	323.49984123	806.83373172
Pbca	236.07845661	288.22595856	196.54822284	333.59548780	810.53204182
Pbcn	239.04926073	296.67104644	203.95867868	338.30521904	817.94558269
$Pna2_1$	236.84019446	281.92447152	196.62248822	335.95555490	817.16542473

Table S5. Calculated crystal cell parameters of the compounds under investigation with the lowest total energies.

Species	Space Groups	a	b	c	α	β	γ
**1a**	$P2_12_12_1$	19.68	29.84	11.66	90.0	90.0	90.0
**1b**	*Pna21*	30.56	21.22	12.45	90.0	90.0	90.0
**1c**	$P\bar{1}$	13.45	16.94	31.47	103.9	104.3	90.6
**1d**	*C2/c*	14.85	16.70	81.47	90.0	84.3	90.0
**1e**	$P2_1/c$	8.27	26.21	60.76	90.0	117.1	90.0
**2a**	$P2_1$	17.32	22.51	8.89	90.0	84.0	90.0
**2b**	$P2_1$	19.33	8.79	25.79	90.0	64.3	90.0
**2c**	*Cc*	22.49	22.19	14.51	90.0	91.4	90.0
**2d**	$P2_12_12_1$	14.24	18.56	29.07	90.0	90.0	90.0
**2e**	$P\bar{1}$	16.76	15.09	18.30	105.0	92.4	96.4

Table S6. The center-center distance and the corresponding hole and electron coupling between the dimer in all of the nearest neighbor pathways for **1a**, **1b**, **1d**, **1e**, and **2a**–**2c** with the lowest total energies.

Species	Space Groups	Pathway	Distance (Å)	Electron Coupling (eV)	Hole Coupling (eV)
1a	*P212121*	1	11.664	1.68×10^{-15}	3.44×10^{-15}
		2	11.664	1.68×10^{-15}	3.44×10^{-15}
		3	13.117	-3.70×10^{-3}	-4.70×10^{-3}
		4	10.062	2.40×10^{-3}	5.23×10^{-4}
		5	16.016	2.70×10^{-6}	-6.63×10^{-7}
		6	19.242	-4.71×10^{-6}	4.90×10^{-6}
		7	16.016	2.70×10^{-6}	-6.63×10^{-7}
		8	16.547	1.01×10^{-5}	-1.39×10^{-5}
1b	*Pna21*	1	21.225	-1.28×10^{-5}	2.08×10^{-5}
		2	21.225	-1.28×10^{-5}	2.08×10^{-5}
		3	17.132	-5.20×10^{-4}	6.02×10^{-4}
		4	17.132	-5.20×10^{-4}	6.02×10^{-4}
		5	11.287	2.95×10^{-4}	6.87×10^{-5}
		6	13.827	-4.25×10^{-7}	-4.67×10^{-5}
		7	11.287	2.95×10^{-4}	6.87×10^{-5}
		8	13.827	-4.25×10^{-7}	-4.67×10^{-5}
1d	*C2/c*	1	11.174	4.22×10^{-5}	-1.41×10^{-4}
		2	11.174	4.22×10^{-5}	-1.41×10^{-4}
		3	11.174	1.71×10^{-6}	-4.85×10^{-6}
		4	11.174	1.71×10^{-6}	-4.85×10^{-6}
		5	16.501	-1.00×10^{-2}	-4.50×10^{-3}
		6	18.403	1.60×10^{-5}	-1.65×10^{-4}
1e	*P2₁/c*	1	8.267	1.72×10^{-4}	-9.23×10^{-4}
		2	8.267	1.72×10^{-4}	-9.23×10^{-4}
		3	16.672	-5.65×10^{-6}	6.29×10^{-6}
		4	17.322	-4.86×10^{-5}	-4.99×10^{-5}
		5	16.672	-5.65×10^{-6}	6.29×10^{-6}
		6	17.322	-4.86×10^{-5}	-4.99×10^{-5}
2a	*P2₁*	1	17.321	9.26×10^{-6}	-3.14×10^{-6}
		2	17.321	9.26×10^{-6}	-3.14×10^{-6}
		3	8.892	-3.90×10^{-3}	-0.0046×10^{-5}
		4	8.892	-3.90×10^{-3}	-0.0046×10^{-5}
		5	12.644	-1.90×10^{-5}	5.44×10^{-5}
		6	12.644	-1.90×10^{-5}	5.44×10^{-5}
		7	12.644	-1.90×10^{-5}	5.44×10^{-5}
		8	12.644	-1.90×10^{-5}	5.44×10^{-5}
2b	*P2₁*	1	8.786	2.95×10^{-4}	0.0028×10^{-5}
		2	8.786	2.95×10^{-4}	0.0028×10^{-5}
		3	19.334	-2.11×10^{-7}	-1.44×10^{-6}
		4	19.334	-2.11×10^{-7}	-1.44×10^{-6}
		5	15.048	4.99×10^{-5}	-3.35×10^{-5}

Table S6. *Cont.*

Species	Space Groups	Pathway	Distance (Å)	Electron Coupling (eV)	Hole Coupling (eV)
		6	16.987	-2.05×10^{-16}	-3.51×10^{-17}
		7	20.265	-8.51×10^{-18}	-4.14×10^{-17}
		8	12.535	2.44×10^{-4}	-1.55×10^{-4}
2c	*Cc*	1	15.797	5.06×10^{-6}	-1.27×10^{-5}
		2	15.797	5.06×10^{-6}	-1.27×10^{-5}
		3	15.797	5.06×10^{-6}	-1.27×10^{-5}
		4	15.797	5.06×10^{-6}	-1.27×10^{-5}
		5	13.349	2.25×10^{-5}	1.33×10^{-5}
		6	13.640	7.91×10^{-4}	-2.42×10^{-4}
		7	11.853	-1.02×10^{-4}	-1.23×10^{-4}
		8	13.349	2.25×10^{-5}	1.33×10^{-5}
2d	*P212121*	1	14.244	-1.34×10^{-5}	3.68×10^{-5}
		2	14.244	-1.34×10^{-5}	3.68×10^{-5}
		3	18.556	6.35×10^{-5}	-8.15×10^{-5}
		4	18.556	6.35×10^{-5}	-8.15×10^{-5}
		5	14.345	-2.45×10^{-4}	3.76×10^{-4}
		6	14.391	-5.48×10^{-6}	-1.67×10^{-6}
		7	11.297	1.29×10^{-4}	1.54×10^{-4}
		8	11.297	1.29×10^{-4}	1.54×10^{-4}
2e	$P\bar{1}$	1	16.762	2.17×10^{-5}	1.10×10^{-4}
		2	16.762	2.17×10^{-5}	1.10×10^{-4}
		3	19.019	6.73×10^{-6}	1.20×10^{-5}
		4	8.057	-3.31×10^{-4}	-6.33×10^{-4}
		5	14.962	2.52×10^{-4}	7.35×10^{-4}
		6	8.368	2.59×10^{-4}	4.53×10^{-4}
